# Nano-organization of synaptic calcium signaling

**DOI:** 10.1042/BST20231385

**Published:** 2024-05-16

**Authors:** Clara I. McCarthy, Ege T. Kavalali

**Affiliations:** 1 Department of Pharmacology, Vanderbilt University, Nashville, TN 37240-7933, U.S.A.; 2 Vanderbilt Brain Institute, Vanderbilt University, Nashville, TN 37240-7933, U.S.A.

**Keywords:** calcium signaling, synapse, synaptic plasticity

## Abstract

Recent studies suggest an exquisite structural nano-organization within single synapses, where sites of evoked fusion — marked by clustering of synaptic vesicles, active zone proteins and voltage-gated calcium channels — are directly juxtaposed to postsynaptic receptor clusters within nanocolumns. This direct nanometer scale alignment between presynaptic fusion apparatus and postsynaptic receptors is thought to ensure the fidelity of synaptic signaling and possibly allow multiple distinct signals to occur without interference from each other within a single active zone. The functional specificity of this organization is made possible by the inherent nano-organization of calcium signals, where all the different calcium sources such as voltage-gated calcium channels, intracellular stores and store-operated calcium entry have dedicated local targets within their nanodomain to ensure precision of action. Here, we discuss synaptic nano-organization from the perspective of calcium signals, where some of the principal findings from early work in the 1980s continue to inspire current studies that exploit new genetic tools and super-resolution imaging technologies.

## Introduction

Calcium's story in cell biology begins with a lucky mistake. In 1882, Sydney Ringer published his findings on the ionic requirements for cardiac contraction [1]. But when only a year later he failed to reproduce these results, he discovered that his eponymous saline solution had been originally prepared with pipe water instead of distilled water [2]. And, as we all know today, calcium was the key missing ingredient for success. From this moment forward, studies demonstrated that calcium ions elicit many intracellular signals that control several cellular functions, and neurons are no exception. If anything, the complex morphology of neurons extends the range of calcium-controlled mechanisms that are fine-tuned within the different compartments of neurons [3]. Broadly, in presynaptic terminals calcium influx triggers fusion of synaptic vesicles and release of neurotransmitters, while transient rises in postsynaptic calcium levels initiate many activity-dependent synaptic plasticity mechanisms and somatic calcium levels regulate gene transcription [4]. These are only a few examples of the many ways in which calcium directly or indirectly regulates a plethora of functions that are critical for neuronal network activity. So how does the same ubiquitous second messenger regulate such a diversity of independent pathways simultaneously in a single cell?

At rest, neurons have an intracellular calcium concentration of 50–100 nM that can locally and rapidly increase up to 1000-fold in nanodomains during activity [4,5]. These calcium fluctuations need to be constantly regulated by the balance between multiple plasma membrane sources of calcium influx and efflux, release and uptake from internal stores and the presence of immobile and diffusive calcium buffers. In many circumstances, calcium influx through one type of calcium permeable channel can selectively control the activity of one specific calcium-dependent protein. This specificity is achieved through tight temporal and spatial control of calcium transients [6]. Calcium signals that occur in neurons act at very different temporal resolutions: from neurotransmitter release in the range of hundreds of microseconds, to gene transcription calcium waves that last minutes to hours. Moreover, the physical distance between the calcium source and their molecular targets strongly determines the efficacy of these calcium signals. Traditionally, mathematical and computational models have been particularly helpful to understand how all the different molecular players affect calcium dynamics at the nanoscale to trigger higher level neuronal functions, such as neurotransmitter release and dendritic summation [7]. On the experimental side, several different calcium indicators have been developed over the years, including synthetic calcium dyes like Quin2, fura-2 and fluo-4 [8], and more recently, protein based genetically encoded calcium indicators, such as GCaMP [9,10]. Since the properties of different neuronal calcium signals vary greatly in amplitude, time course and frequency, it is important to understand the intrinsic properties of the different imaging probes (sensitivity, affinity, brightness, kinetics and dynamic range) [9]. Moreover, while wide-field microscopy imaging devices are appropriate for *in vitro* imaging, confocal and two-photon microscopy is better suited to image neurons that are located deeper in the brain tissue [6]. In the past decade, development of new and more efficient calcium indicators and high-resolution imaging technologies allowed scientists to measure calcium transients with a higher spatial and temporal resolution [11,12]. Here, we will discuss the recent findings into the nanoscale spatial specificity of calcium signals in the regulation of synaptic function.

## From multiple calcium sources arise multiple nanodomains

The notion that calcium signals act in a highly temporally and spatially specific manner at the nanoscale predates current notions on structural nano-organization of synapses [13]. Indeed, the nanodomain action of calcium signals strongly supports the premise that there needs to be strict control of the distance between calcium sources and their targets to achieve the functional specificity of nanocolumns within single synapses.

The first of many ‘calcium nanodomain’ computational models, published in 1984, proposed that the opening of individual calcium channels could create discrete spatial domains in the nanometer range, where calcium concentrations were significantly high locally but dropped down rapidly with distance away from the channel pore [14]. The calcium nanodomain model proposes three key benefits for neurons: (1) the influx of just a few calcium ions rapidly increases the local intracellular calcium concentration to the micromolar level necessary to activate calcium sensors, (2) this local rise in the calcium level rapidly disperses by ion diffusion and calcium buffers, and (3) multiple calcium signaling pathways occur without interference from each other in the same neuronal compartment. In some situations, calcium domains can overlap creating microdomains, which can additionally summate producing radial calcium gradients [15,16]. Evidence indicates that these spatiotemporal patterns of calcium dynamics are crucial for synapse specificity. For example, calcium nanodomain signaling is prevalent at presynaptic terminals that rapidly release neurotransmitters, while radial gradients are thought to be important in presynaptic terminals that slowly release neuropeptides [16]. To elucidate how these nanoscale calcium dynamics are regulated, it is necessary to understand how each of the key molecular players contribute to calcium signals at the synapse.

**Figure 1.**
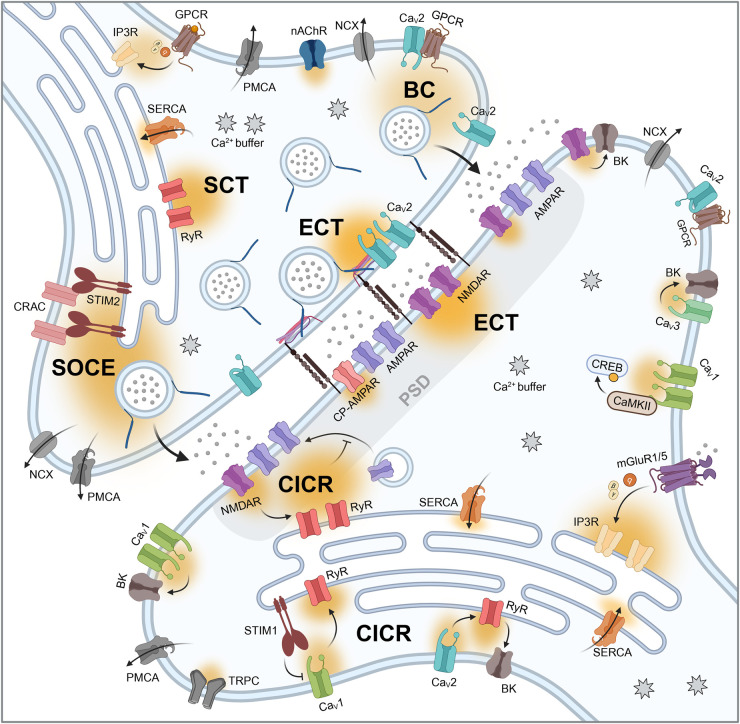
Nano-organization of calcium signals in the excitatory synapse. The arrival of an action potential to the presynaptic terminal triggers the opening of clustered Ca_V_2 channels, producing evoked calcium transients (ECT) that induce synaptic vesicle fusion and evoked neurotransmitter release. In contrast, the stochastic opening of diffusely distributed Ca_V_2 channels contributes to the baseline calcium (BC) signal that is partially responsible for spontaneous neurotransmitter release. Another calcium signal that influences spontaneous neurotransmission is the store-operated calcium entry (SOCE), where STIM2 proteins from the ER activate CRAC channels in the plasma membrane. Meanwhile, RyR in the presynaptic ER produce spontaneous calcium transients (SCT) that are independent of neurotransmitter release. In the postsynaptic density (PSD), evoked neurotransmitter release activates clustered NMDARs and Ca^2+^-permeable AMPARs to produce postsynaptic evoked calcium transients (ECT). Moreover, spontaneous release of glutamate activates NMDARs and this triggers RyR-mediated calcium-induced calcium release (CICR) from the ER that inhibits AMPAR insertion in the plasma membrane. Other forms of CICR in the postsynaptic compartment are triggered by Ca_V_1 and Ca_V_2 channels. Finally, GPCRs, like mGluR1/5, can trigger Ca^2+^ ER release through IP3R activation. All of these calcium signals are tightly controlled by the presence of Ca^2+^ buffers (mobile or immobile) and Ca^2+^ pumps (cell surface or ER membrane). AMPAR, α-amino-3-hydroxy-5-methyl-4-isoxazolepropionic acid receptor; BC, baseline calcium; BK, big potassium channel; CaMKII, calcium/calmodulin-stimulated protein kinase II; CP-AMPAR, Ca^2+^-permeable AMPAR; Ca_V_1, voltage-gated calcium channel type 1; Ca_V_2, voltage-gated calcium channel type 2; Ca_V_3, voltage-gated calcium channel type 3; CICR, calcium-induced calcium release; CRAC, calcium release activated channel; CREB, cAMP response element-binding protein; ECT, evoked calcium transient; GPCR, G-protein coupled receptor; IP3R, inositol triphosphate receptor; mGluR1/5, metabotropic glutamate receptor type 1/5; nAChR, nicotinic acetylcholine receptor; NCX, sodium-calcium exchanger; NMDAR, *N*-methyl-d-aspartate receptor; PMCA, plasma membrane Ca^2+^-ATPase; PSD, postsynaptic density; RyR, ryanodine receptor; SCT, spontaneous calcium transient; SERCA, sarcoplasmic reticulum Ca^2+^-ATPase; SOCE, store-operated calcium entry; STIM1/2, stromal interaction molecule 1/2; TRPC, transient receptor potential canonical channel.

Calcium ions can flow inward from extracellular spaces through voltage- or ligand-gated channels and they can be released from intracellular organelles (Figure 1). In neurons, calcium signals arise from different sources, including voltage-gated calcium (Ca_V_) channels, *N*-methyl-d-aspartate receptors (NMDAR), calcium-permeable α-amino-3-hydroxy-5-methyl-4-isoxazolepropionic acid receptors (AMPAR) and the endoplasmic reticulum (ER). In particular, neurons express several subtypes of Ca_V_ channels with very distinct structures, biophysical properties, pharmacology and physiological functions. The Ca_V_1 subfamily encodes three different neuronal channels (Ca_V_1.2, Ca_V_1.3 and Ca_V_1.4, formerly called L-type) that display slow voltage-dependent gating characteristics and typically a somatodendritic distribution. Instead, the high voltage-activated Ca_V_2 channels (Ca_V_2.1, Ca_V_2.2 and Ca_V_2.3, formerly called P/Q-, N- and R-type) are canonically expressed in presynaptic membranes. Lastly, Ca_V_3 channels (Ca_V_3.1, Ca_V_3.2 and Ca_V_3.3, formerly called T-type) are activated by lower voltages and usually found in postsynaptic membranes. Additionally, mitochondria can act as calcium buffers, taking up calcium through the calcium uniporter and then slowly releasing it to the cytosol through sodium-calcium exchanger [17]. Additional sources of extracellular calcium include transient receptor potential type C (TRPC) channels and nicotinic acetylcholine receptors, among others [18,19]. These multiple calcium sources are strictly orchestrated with spatial and temporal specificity. Calcium buffers (including calcium binding proteins that do not necessarily activate any pathway) and transmembrane pumps, like the sodium-calcium exchanger and the plasma membrane Ca^2+^-ATPase, limit the amplitude, duration and spatial extent of calcium transients and keep resting calcium below 100 nM [20]. In the following sections, we will further address the specific functions and spatial regulation of the key calcium sources in the pre- and postsynaptic compartments.

## Calcium signals at presynaptic terminals

### Presynaptic voltage-gated calcium channels

**Figure 2.**
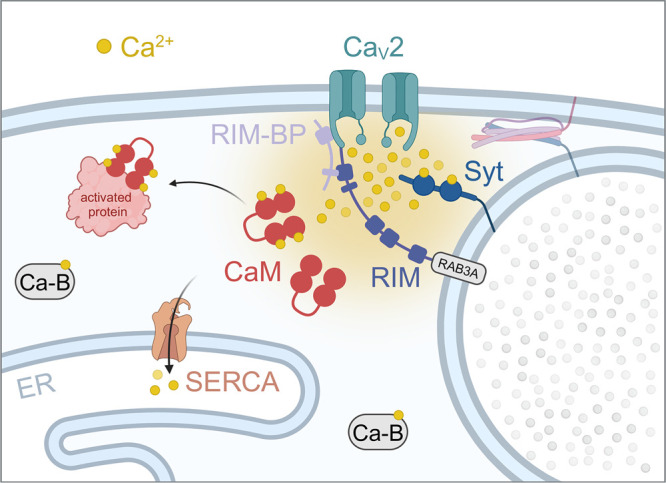
Regulation of local calcium concentration in the nano- and microdomain of a presynaptic Ca_V_ channel. The opening of presynaptic Ca_V_2 channels causes a fast and transient calcium (Ca^2+^) influx. Calcium concentration rapidly increases up to 50–100 µM in the Ca_V_2 channel's nanodomain, activating closely located targets like sytaptotagmin (Syt). The scaffolding proteins RIM and RIM-BP tether Ca_V_2 channels to synaptic vesicles, granting that synaptotagmin molecules are at the nanodomain reach. Calcium ions bind to synaptotagmin and trigger vesicle fusion and neurotransmitter release. In the channel's nanodomain, calcium concentration is modulated by calmodulin (CaM), which binds calcium and activates specific downstream target proteins. Conversely, calcium oscillations in the micrometer range are buffered by other mobile calcium binding proteins (Ca-B) and calcium pumps, such as the sarcoplasmic reticulum Ca^2+^-ATPase (SERCA).

In the presynaptic terminal, neurotransmitter release is predominantly triggered by calcium ion influx through the Ca_V_2 channel subfamily [21,22]. Increasing experimental and mathematical evidence supports that the cooperative action of at least five calcium ions entering the presynaptic terminal through just a few or possibly even one open Ca_V_ channel can trigger release of excitatory and inhibitory neurotransmitters [5,23–25]. At rest, synaptic vesicles can form interactions with several SNARE complexes and the activation of at least three of these complexes is needed to trigger release [26]. Considering that each SNARE complex can bind up to two synaptotagmin-1 molecules [27] and that each of these requires binding of two calcium ions to activate [28], neurotransmitter release under these conditions would involve ∼12 calcium ions. Although the exact number of calcium ions needed to trigger vesicular fusion is still unknown and most likely variable between different synapses, it is clear that a small influx of calcium is sufficient to elicit fast synchronous release (Figure 2). In this sense, release triggered by a single calcium nanodomain would not only maintain a low signal-to-noise ratio and avoid the toxic effects of high intracellular calcium levels, but it would additionally reduce the energetic demands necessary for ion extrusion [29]. For instance, a recent study performing cell-attached patch clamp recordings and lattice-light microscopy from single lamprey giant axon active zones demonstrated that presynaptic Ca_V_ channels form nanodomains with primed vesicles with a 1:1 stoichiometry and that calcium entry occurs through one or a few open channels localized closely to primed vesicles [30]. A previous study using a combination of experimental data from frog neuromuscular junction and computational modeling showed that, on average, only two to six Ca_V_2 channels open in response to an action potential at each active zone. The finding that a single Ca_V_2 channel could trigger release implies that the channel and the synaptic vesicle must be closely linked by a tether (Figure 2) [31].

The spatial organization of the active zone determines whether one or more Ca_V_2 open channels are needed to cause synaptic vesicle fusion. The distance between Ca_V_2 channels and the synaptic vesicles needs to be within ∼20 nm to trigger neurotransmitter release [32]. It was predicted that a synaptic vesicle located 30 nm from an open Ca_V_ channel would have a release probability of 0.3, whereas at 50 nm from the pore opening the release probability would be 0.1 [29]. Moreover, clusters integrated by Ca_V_2 channels, synaptic vesicles and SNARE proteins in the active zone can associate with proteins across the synaptic cleft and the postsynaptic density (Figure 1). These trans-synaptic alignments are referred to as nanocolumns and they are thought to facilitate synchronous evoked neurotransmitter release [33]. Meanwhile, the highly structured fly neuromuscular junction has proved to be a useful model to study the relationship between the presynaptic nanostructure and neurotransmission. A recent study combined functional quantal analysis of neurotransmitter release from live imaging data with direct stochastic optical reconstruction microscopy (dSTORM) super-resolution molecular imaging in the same active zones from the fly neuromuscular junction [34]. They found that neurotransmitter release dynamics strongly depend on the nanoscale localization and density of calcium channels, complexin and the scaffolding protein Bruchpilot in the fly neuromuscular junction synapses. Interestingly, some studies show that this clustered structure of Ca_V_2 channels and active zone proteins can be quite dynamic under specific conditions. Tracking experiments in hippocampal synapses showed that ∼60% of Ca_V_2.1 and Ca_V_2.2 channels were mobile within the active zone, while confined to this presynaptic compartment [35]. This dynamic channel density was calcium-dependent and it was predicted to determine channel cooperativity and influence release probability and short-term plasticity [35,36].

In addition to controlling action potential-evoked neurotransmitter release, presynaptic Ca_V_2 channels contribute to spontaneous neurotransmission, although it is not fully understood how and to what extent. Some studies suggest that the Ca_V_2 channels that impact spontaneous release are distant from synaptic vesicle sensors [37,38]. On the other hand, there is also evidence pointing towards both evoked and spontaneous release originating from the same clustered calcium channels microdomains [39]. Moreover, a study comparing spontaneous release throughout different synapses observed that spontaneous neurotransmission strongly depends on presynaptic Ca_V_2 channels in synapses with tight coupling between channels and synaptic vesicles, whereas Ca_V_2-dependence of spontaneous release was more variable in loosely coupled synapses [40]. Recently, Wang et al. [41] showed that a subpopulation of Ca_V_2 channels contribute to presynaptic baseline calcium levels and facilitate spontaneous neurotransmitter release. Using a genetically encoded calcium indicator linked to synaptobrevin-2 (GCaMP8s-Syb2), they identified three distinct calcium signals deriving from three non-overlapping calcium sources within presynaptic boutons. First, the synchronized opening of Ca_V_2 channels in response to depolarization was responsible for calcium transients that led to evoked neurotransmitter release. In contrast, the stochastic single-channel opening of more diffusely distributed Ca_V_2 channels contributed to the baseline calcium levels in presynaptic boutons and was partially responsible for excitatory spontaneous neurotransmission (Figure 1). Interestingly, an experimental strategy based on activity-dependent photobleaching of the GCaMP8s-Syb2 probe demonstrated that the evoked Ca_V_2-mediated calcium transients are spatially segregated from scattered Ca_V_2 channels that contribute to baseline calcium and spontaneous release [41] (Figure 1). Finally, they detected spontaneous calcium events that originated from ER release sites (which we will discuss in the following section). These results show that non-overlapping calcium signals impact distinct forms of neurotransmission and are consistent with earlier studies suggesting that distinct vesicle pools occur in spatially different regions of the presynaptic terminal [42–44]. But what are the molecular mechanisms that lead to this strict spatial segregation?

Presynaptic Ca_V_2 channels interact with multiple protein complexes to form the core of the active zone. A key quantitative proteomic analysis reported that there are over 200 proteins in the Ca_V_2 channel nano-environment that directly or indirectly interact with them [45]. Ca_V_2 channels form heterogeneous complexes with several proteins that either anchor or disrupt the channels to the active zones and link them to release machinery, such as RIM, RIM-BP, syntaxin-1, SNAP-25, synaptotagmin-1, Munc13 and CASK [46]. For example, RIM tethers Ca_V_2 channels to actives zones either by association with the Ca_V_β auxiliary subunit, by association with the pore-forming Ca_V_α1 subunit or indirectly by interacting with RIM-BP. Moreover, some of these interactions are Ca_V_2 subtype specific, allowing each subtype to have a different functional nano-environment at the synaptic terminal. In the neuromuscular junction, the RIM-BP-Bassoon interaction specifically up-regulates the synaptic localization of Ca_V_2.1 and interfering with this interaction significantly impairs evoked neurotransmission [47,48]. Since Ca_V_2 channel subtypes and their splice isoforms have very different current kinetics, this spatial segregation will have an impact on synaptic fusion, although it is not yet fully understood to what extent [49,50]. Adding further complexity, Ca_V_2.1 and Ca_V_2.2 channels have been shown to interact with proteins of the cytoskeleton (ankyrin B, dynamin-1, microtubule-associated proteins), G-protein coupled receptors (GPCRs) (mGluR1, D1R, D2R, MT1, etc.) and lipid rafts [46,51]. The spatial localization of presynaptic Ca_V_ channels and the contribution of the specific Ca_V_2 subtypes would therefore be determined by arranging all these physical interactions, considering the specific protein expression in different neuronal subtypes and the competing affinities of these multiple physical interactions. There is still much to uncover regarding the mechanisms by which specific subtypes of Ca_V_ channels and splice isoforms are trafficked and arranged within the active zone.

### ER calcium modulation at presynaptic terminals

The ER in neurons is a complex structure that is often contiguous across the whole cell, from the soma to distal regions of dendrites and axon and integrates the activity of these autonomous compartments. The ER behaves both as a calcium sink and as a calcium store, clearing or releasing calcium and maintaining calcium level homeostasis [52]. Moreover, release from the ER can generate calcium transients that trigger specific signaling pathways and modulate synaptic function. Multiple pathways can evoke calcium release from the ER, including the inositol triphosphate receptor (IP3R) pathway and the ryanodine receptor (RyR) pathway. For instance, activation of Gq-coupled GPCRs can cause IP3R-mediated calcium release, by activation of phospholipase C and IP3 synthesis [53]. Alternatively, cytosolic calcium increase can activate RyRs in the ER membrane and evoke calcium-induced calcium release (CICR) [54].

Although most studies focused on the postsynaptic ER calcium release functions, computational and experimental data show that the ER plays an important role at the presynaptic site. Using a hippocampal neuron computational model, Singh and collaborators [55] predicted that presynaptic ER is critical for short-term synaptic plasticity and for the maintenance of low release probability synapses. Moreover, in hippocampal presynaptic terminals, a partial and transient depletion of ER calcium leads to an increase in basal presynaptic calcium levels, as a result of store-operated calcium entry (SOCE) mediated by the stromal interaction molecule STIM2 [56] (Figure 1). Interestingly, this SOCE activation mechanism enhances glutamatergic spontaneous neurotransmission through the calcium sensor synaptotagmin7 and contributes to the alterations in neurotransmission observed during chronic ER stress. In contrast with this ER-dependent bulk increase in presynaptic calcium, a following study identified spontaneous and transient calcium sparks originated by the ER at presynaptic terminals [41]. These spontaneous calcium events were mediated by RyR and were completely independent on spontaneous neurotransmitter release (Figure 1). Further experiments revealed that spontaneous ER calcium events were spatially separate from Ca_V_2 channels calcium signals associated with synaptic vesicle release in the presynaptic bouton [41]. Taken together, these results demonstrate that the presynaptic ER can exhibit multiple calcium signals that are distinct in their molecular nature, in their kinetics and that differentially impact synaptic neurotransmission.

## Calcium signals at postsynaptic sites

### Postsynaptic voltage-gated calcium channels

Calcium channels of the Ca_V_1 subfamily form signaling complexes on postsynaptic membranes. Ca_V_1.2 and Ca_V_1.3 channels abound in synaptic spines, dendritic shafts and neuronal cell bodies [57,58]. The low-voltage activated Ca_V_3 channels are also present in postsynaptic membranes, although they are mainly concentrated in the axon initial segment where they control excitability and firing patterns [59,60]. The opening of somatodendritic Ca_V_1 channels generates membrane depolarizations called calcium potentials and calcium waves that contribute to signal propagation and promote transcription and calcium-dependent plasticity, such as long-term potentiation (LTP) [61,62]. The heterogeneous dendritic spine morphology and the tightly regulated channel distribution are two crucial factors that govern the spatial range of these postsynaptic calcium signals [3]. Interestingly, Ca_V_2 channels are also found in somatodendritic compartments [63–68], although their functional roles in postsynaptic neuronal signaling are not widely explored.

Postsynaptic Ca_V_1 channel signaling complexes regulate channel function and synaptic plasticity. For instance, Ca_V_1.3 channels form functional clusters of two or more channels along the surface membrane of hippocampal neurons [69,70]. Cooperative gating of these clustered Ca_V_1.3 channels facilitates calcium currents and increases firing rates in hippocampal neurons [69]. Moreover, physical interactions can promote functional coupling between the channels and their targets and enhance the signaling efficacy. Thanks to targeted Ca_V_1.3 clustering, a small increase in the channel's local calcium nanodomain can increase the phosphorylation of the nuclear transcription factor CREB in hippocampal neurons, avoiding the need to increase bulk calcium [71]. Similarly, direct interaction between Ca_V_1.2 channels and CaMKII facilitates downstream calcium-regulated protein kinases that activate CREB and other transcription factors [72] (Figure 1). The calcium-regulated phosphatase calcineurin also binds to Ca_V_1.2 channels. Thus, calcium influx through Ca_V_1.2 activates calcineurin and triggers dephosphorylation of transcription factors that diffuse into the nucleus and activate gene transcription [73–75]. In contrast, other physical interactions modulate Ca_V_1 channel function. For example, interaction between β2-adrenergic receptors with Ca_V_1.2 regulates channel activity and LTP induction [76,77].

In addition, functional coupling of Ca_V_ channels with different potassium channels occurs frequently at postsynaptic sites. At ER contact sites with the plasma membrane, Ca_V_1 channels physically interact with type 2 voltage-gated potassium channels (K_V_2) to prevent calcium neurotoxicity, and disruption of this interaction drives neuronal death [78]. Similarly, low-voltage activated Ca_V_3 channels interact with several potassium channel subtypes. In cerebellar inhibitory interneurons, Ca_V_3 channels form signaling complexes with K_V_4 channels, allowing these potassium channels to function in the subthreshold membrane potential range and regulate neuronal firing properties [79]. Meanwhile, several studies demonstrated that Ca_V_ channels are functionally coupled with the calcium-activated ‘big’ potassium (BK) channels, especially in the case of Ca_V_1.2 and Ca_V_3.1 channels, and less frequently with Ca_V_2.1 and Ca_V_2.2 [46] (Figure 1). BK channels are activated by both voltage and an increase in intracellular calcium concentration. Since the calcium concentration required for BK activation is ≥10 µM, these channels need to be spatially close to the source of calcium influx [80]. For example, in inhibitory interneurons from the dorsal cochlear nucleus, Ca_V_2.1-RyR-BK channels form a double calcium nanodomain coupling that controls firing bursts [81]. Thus, the opening of somatic Ca_V_2.1 channels triggers RyRs-induced calcium release from the ER, which in turn activates closely located BK channels (Figure 1).

### Postsynaptic glutamate receptors that contribute calcium entry

The NMDA-sensitive ionotropic receptors bind glutamate and mediate a significant part of calcium entry into dendritic spines that is key for long-term modification of synaptic strength. These receptors act as coincidence detectors of glutamate availability and postsynaptic membrane depolarization. The simultaneous occurrence of both stimuli overcomes the magnesium block of these channels and triggers the opening of NMDARs, which results in a non-specific inward current of cations. Approximately 6–12% of this current corresponds to calcium ions [82–84]. This relatively high permeability to calcium ions confers NMDARs a central role in synaptic plasticity under physiological conditions and neuronal death under excitotoxic pathological conditions.

The specific subunit composition of NMDARs and their phosphorylation level are two key factors that regulate NMDAR function. For instance, CA1 hippocampal neurons can preferentially express different combinations of NMDAR subunits, altering the receptor's permeability to calcium ions [85]. A more dynamic way of controlling NMDAR permeability to calcium is the phosphorylation level, where increased phosphorylation increases calcium influx through NMDAR and dephosphorylation of these receptors decreases their calcium permeability [86]. Additionally, evidence shows that NMDAR are functionally coupled with other molecular players in a nanoscale environment. In pyramidal neurons, calcium influx through NMDAR activates BK channels specifically at small-headed dendritic spines with narrow necks, locally suppressing excitatory postsynaptic potentials [80] (Figure 1). Another evidence for NMDA-BK coupling comes from a study in cortical pyramidal neurons, where local calcium entry through dendritic NMDARs activates closely located BK channels, resulting in hyperpolarization and increasing the threshold for LTP [87]. In this manner, dendritic compartmentalization and nanoscale proximity between proteins allow small calcium influxes through NMDARs to fine tune synaptic responses.

Despite the well-established models on NMDAR function's dependence on membrane depolarization, there is also substantial evidence that NMDARs can function under resting conditions and trigger calcium signaling even when magnesium is partially blocking their pores [88,89]. This resting calcium signaling by NMDARs is attributed to the fact that magnesium block of NMDARs is likely incomplete even at low membrane potentials. Thus, the residual calcium influx is sufficient to trigger CICR that activates high affinity calcium-calmodulin dependent targets such as eEF2 kinase and ultimately regulates dendritic protein translation [89] (Figure 1). Even though spontaneous and evoked glutamate release driven NMDAR-mediated calcium transients often occur at the same synapse, use-dependent block of NMDAR calcium signals revealed a sub-synaptic separation of these two modes of neurotransmission [44].

Furthermore, calcium permeable AMPA receptors, characterized by the presence of GluA1 and lack of the GluA2 subunit, are another class of ionotropic glutamate receptors. Because of their remarkably fast kinetics, AMPAR-dependent calcium transients contribute to highly localized dendritic calcium signals, enabling calcium compartmentalization even in neurons lacking dendritic spines [90]. In addition to a higher amplitude calcium influx, calcium permeable-AMPARs-containing neurons can show alterations in sodium and potassium homeostasis and a higher vulnerability to glutamate excitotoxicity, compared with neurons lacking calcium permeable-AMPARs [91].These receptors are differentially expressed in some neuronal subtypes, like neocortical pyramidal neurons and aspiny GABAergic neurons [92,93]. The AMPAR subunit composition varies in a synapse-specific manner within individual neurons and is it dynamically regulated in response to neuronal activity, triggering LTP induction [94,95]. Conversely, LTP induction increases functional expression of calcium permeable-AMPARs, reaching more than twice the basal expression 30 min after LTP induction [96]. Moreover, calcium permeable-AMPARs in interneurons from the nucleus accumbens contribute to endocannabinoid-mediated long-term depression (LTD) of glutamate release, ultimately affecting basal locomotion [97]. Changes in calcium permeable-AMPARs have been associated with Alzheimer's disease, substance abuse disorders, ischemic stroke, oxidative stress and neurodegeneration [98–101]. In recent years, there have been increasing efforts to uncover the intracellular mechanisms that regulate calcium permeable-AMPAR expression, especially in the context of neuropsychiatric disorders. For instance, the A-kinase anchoring protein scaffolds kinases and phosphatases to regulate GluA1 phosphorylation and trafficking [102]. On the other hand, inflammatory pain increases GluA1 expression in the spinal cord dorsal horn and the increased number of synaptic GluA1 nanoclusters correlated with pain-related responses [103].

### Postsynaptic ER calcium modulation

The ER extends through the entire neuron, reaching even the most distal dendrites. As mentioned above, it acts as calcium sink and removes the excess in calcium caused by neuronal activity via the sarcoendoplasmic reticulum calcium transport ATPase (SERCA). Simultaneously, the ER functions as a calcium source, releasing calcium to the cytosolic compartment. ER-mediated calcium oscillations have been implicated in dendritic spine morphology and maturation, facilitation of AMPARs, regulation of AMPAR trafficking, inhibition of NMDARs and of Ca_V_ channels and induction of Hebbian plasticity [52]. In particular, depletion of postsynaptic ER calcium activates STIM proteins, which then aggregate at ER-plasma membrane junctions and trigger the clustering and opening of calcium release-activated channels (CRACs) [104]. Among many functions attributed to the two leading ER-release pathways, RyR-mediated calcium release preferentially contributes to amplify fast calcium sparks in dendrites, while IP3R-mediated calcium release leads to long-lasting signals and contribute to dendritic calcium waves [105]. Moreover, calcium release via IP3Rs enhances postsynaptic responses and unsilences synapses in hippocampal neurons [106]. Although both ER-release pathways ultimately modulate LTP and LTD plasticity, their proposed intracellular mechanisms are quite different [107].

As indicated above, even small calcium influxes through NMDARs or Ca_V_1 channels activate RyR-mediated CICR from the ER. In hippocampal neurons, spontaneous release of glutamate activates NMDARs near the postsynaptic density, even when these receptors are partially blocked by magnesium ions [44,89]. This small influx of calcium activates RyRs and triggers CICR from the ER, which amplifies the signal. At rest, these calcium transients block AMPAR synthesis to maintain homeostatic neurotransmission [89] (Figure 1). On the other hand, Ca_V_1 channels at the cell surface form signaling complexes with RyRs in the ER to promote CIRC (Figure 1). The close proximity facilitates RyR activation with only a small calcium influx through Ca_V_1 channels and it is restricted by the interaction of STIM1 that induces Ca_V_1 channel internalization [108,109]. CICR causes a strong depletion of the dendritic ER calcium reservoir within less than tens of milliseconds and the calcium replenishment mechanisms that follow are subjected to the spine's nanoscale organization. Experimental and computational data demonstrate that ER calcium replenishment occurs via STIM1-CRAC pathways and SERCA pumps very closely located to the plasma membrane [110]. In contrast, increasing distance between the SERCA pumps and the plasma membrane would increase the chance of calcium ions to activate RyR and cause ER calcium depletion [110]. Overall, these results highlight the importance of the nanoscale spatial organization of the ER and its calcium-permeable channels within dendritic spines.

Finally, Gq-coupled GPCRs present in the postsynaptic membrane, such as metabotropic glutamate receptors (mGluRs), indirectly contribute to calcium signals at postsynaptic sites. The subfamily of mGluRs group I typically trigger the Gq signaling pathway that generates IP3 and activates IP3R-dependent calcium release from the ER stores [111] (Figure 1). Calcium signals induced by group I mGluRs often lead to cell depolarization, increases in neuronal excitability and induction of LTP or LTD plasticity, however spatial and temporal contribution of mGluR signaling to calcium transients at the nanoscale remain poorly understood.

## Spatial control by calcium buffers

The distribution of calcium buffers inside neurons shapes the diffusion of calcium, as the buffers rapidly bind to free calcium ions without activating any signaling pathway. The spatial organization of mobile and immobile buffers offers neurons a broad range of options for modulating calcium domains [112]. For example, immobile buffers play a unique role in shaping calcium gradients since they dramatically slow the spread of a calcium waves in dendrites. In the presynaptic terminal, endogenous calcium buffers very effectively terminate local calcium increases after Ca_V_ channel closure, with an estimated delay of 100 µs [25]. The study of how mobile and immobile calcium buffers affect the time course and spatial range of calcium signals has been approached mainly with computational modeling and to less extent with experimental data. In a 3-dimensional model of dendritic spines, endogenous fixed and mobile calcium buffers had substantial impact on global calcium signals, whereas nanodomain calcium signals driven by Ca_V_ channels and NMDARs clusters were less affected [113]. Moreover, buffering of cytosolic calcium prevented neuronal death in a culture model of Parkinson's disease [114]. Another computational study that contrasted results with experimental data showed that while calcium buffers have a major effect on whole spine calcium signals, they do not demonstrate a significant impact on NMDAR and Ca_V_ channel calcium nanodomains [115]. These nanodomains typically activate calmodulin, which in turn activates numerous downstream signaling proteins (Figure 2) [116]. Thus, calmodulin activation in the NMDAR nanodomains could have different downstream targets than calmodulin in Ca_V_ channel nanodomains [117]. Taken together, these studies suggest that both mobile and immobile calcium buffers act as a low-pass filter for bulk calcium oscillations but show little effect on calcium nanodomains.

## Perspectives

- The tight nanoscale spatial control of calcium signals in the synapse is fundamental for the multiple intracellular pathways triggered by this ubiquitous second messenger.
- The body of evidence discussed here shows that the multiple calcium sources present in the presynaptic and postsynaptic compartments need to be located only nanometers apart from their specific targets. However, the molecular mechanisms that govern this spatial nano-organization are yet unclear.
- The diffraction-limited nature of current live-imaging approaches used to monitor calcium signals strongly limits the spatial resolution that these studies can achieve. Thus, new experimental approaches that combine super-resolution ‘nanoscopy’ and dynamic functional imaging are greatly needed to visualize calcium dynamics within individual presynaptic terminals and postsynaptic sites.

## Abbreviations

AMPAR: α-amino-3-hydroxy-5-methyl-4-isoxazolepropionic acid receptor
BK: big potassium channel
CaMKII: calcium/calmodulin-stimulated protein kinase II
CASK: calcium/calmodulin-dependent serine protein kinase
Ca_V_: voltage-gated calcium channel
CICR: calcium-induced calcium release
CRAC: calcium release activated channel
CREB: cAMP response element-binding protein
D1R/D2R: dopamine receptor type 1/type 2
eEF2: eukaryotic transcription elongation factor
ER: endoplasmic reticulum
GABA: gamma-aminobutyric acid
GCaMP8s: genetically encoded calcium indicator 8s
GPCR: G-protein coupled receptor
IP3R: inositol triphosphate receptor
KV: voltage-gated potassium channel
LTD: long-term depression
LTP: long-term potentiation
mGluR: metabotropic glutamate receptor
MT1: melatonin receptor type 1
NCX: sodium-calcium exchanger
NMDAR: *N*-methyl-d-aspartate receptor
PMCA: plasma membrane Ca^2+^-ATPase
RIM: Rab3-interacting molecule
RIM-BP: Rab3-interacting molecule binding protein
RyR: ryanodine receptor
SERCA: sarcoplasmic reticulum Ca^2+^-ATPase
SNAP-25: synaptosomal associated protein 25
SNARE: soluble *N*-ethylmaleimide-sensitive factor activating protein receptor
SOCE: store-operated calcium entry
STIM1/2: stromal interaction molecule ½
TRPC: transient receptor potential canonical channel
